# THE INTERSECT OF SOCIAL DETERMINANTS, MOTIVATION, AND BLACK WOMEN TRIATHLETES

**DOI:** 10.1093/geroni/igz038.2192

**Published:** 2019-11-08

**Authors:** Candace Brown

**Affiliations:** Duke University, Durham, North Carolina, United States

## Abstract

Social determinants of health are often adjacent to negative impacts on physical health due to the association of a sedentary lifestyle. Yet, knowledge is limited regarding how motivation to exercise, through sport, may relate to social determinants. This study aimed to understand the intersect of social determinants of Black women who exercise, by participating in triathlons, using the novel Motivations for Triathletes Interview Guide (MOTIG). The Organismic Integration Theory guided an assessment of social determinants among (N = 12) Black women from nine scales within the MOTIG. The subtheme ‘physical awareness’ supported Health Orientation while ‘social support’, ‘camaraderie’ and ‘family’, all supported the Affiliation scale. Results indicate that Black women who exercise through triathlon are motivated because certain social determinants are positively addressed. Future interventions directed toward sedentary women should consider how to positively reinforce individual and social determinants to increase motivation to exercise.

